# The Potassium Channel Odyssey: Mechanisms of Traffic and Membrane Arrangement

**DOI:** 10.3390/ijms20030734

**Published:** 2019-02-09

**Authors:** Jesusa Capera, Clara Serrano-Novillo, María Navarro-Pérez, Silvia Cassinelli, Antonio Felipe

**Affiliations:** Molecular Physiology Laboratory, Departament de Bioquímica i Biomedicina Molecular, Institut de Biomedicina (IBUB), Universitat de Barcelona, Avda. Diagonal 643, 08028 Barcelona, Spain; 11jesusa@gmail.com (J.C.); clara.serrano.n@gmail.com (C.S.-N.); navarromarya@gmail.com (M.N.-P.); silvia.cassinelli73@gmail.com (S.C.)

**Keywords:** potassium channels, traffic, auxiliary subunits, forward traffic, retention, organelles, post-translational modification

## Abstract

Ion channels are transmembrane proteins that conduct specific ions across biological membranes. Ion channels are present at the onset of many cellular processes, and their malfunction triggers severe pathologies. Potassium channels (KChs) share a highly conserved signature that is necessary to conduct K^+^ through the pore region. To be functional, KChs require an exquisite regulation of their subcellular location and abundance. A wide repertoire of signatures facilitates the proper targeting of the channel, fine-tuning the balance that determines traffic and location. These signature motifs can be part of the secondary or tertiary structure of the protein and are spread throughout the entire sequence. Furthermore, the association of the pore-forming subunits with different ancillary proteins forms functional complexes. These partners can modulate traffic and activity by adding their own signatures as well as by exposing or masking the existing ones. Post-translational modifications (PTMs) add a further dimension to traffic regulation. Therefore, the fate of a KCh is not fully dependent on a gene sequence but on the balance of many other factors regulating traffic. In this review, we assemble recent evidence contributing to our understanding of the spatial expression of KChs in mammalian cells. We compile specific signatures, PTMs, and associations that govern the destination of a functional channel.

## 1. Introduction

Ion channels are transmembrane (TM) proteins that form hydrophilic pores across the lipid bilayer, driving specific ions down the electrochemical gradient. Potassium channels (KChs) share a highly conserved signature within the pore, known as the selectivity filter, which allows for the selective passage of K^+^. However, the KCh superfamily shows high divergence among the sensing domains. This feature permits KCh gating in response to a wide variety of signals [1].

**Figure 1:**
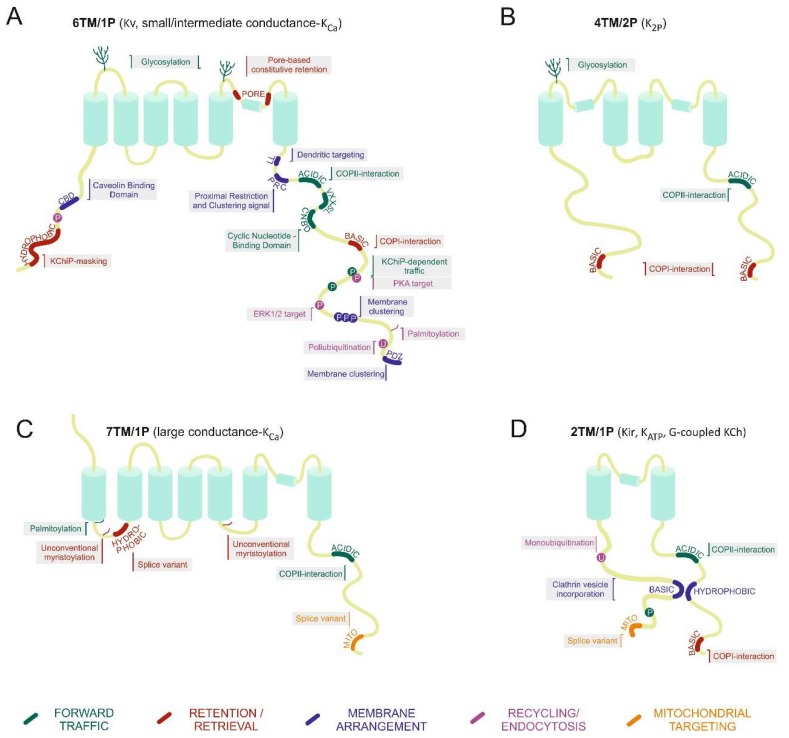
Traffic motifs and molecular interacting signatures within K^+^ channel structures. A schematic representation of each family of potassium channels. (**A**) 6TM/1P; (**B**) 4TM/2P; (**C**) 7TM/1P; and (**D**) 2TM/1P. Structural domains and post-translational modifications (PTMs) affecting traffic and the destination of different channels in each family are shown. Cartoons represent a compilation of signatures documented in each structural family. It is important to highlight that not all signatures are present in the same channel. The color code corresponds to the main dominant effect in traffic. Green, forward traffic. Red, retention/retrieval domains. Blue, membrane arrangement. Magenta, channel recycling and endocytosis. Orange, mitochondrial targeting. Basic, cluster of basic residues; Acidic, cluster of acid residues; Hydrophobic, hydrophobic clusters; LL, di-leucine motif; P, phosphorylation; U, ubiquitination; Mito, mitochondria; PDZ, domains.

From a structural point of view, KChs are classified into four groups (Figure 1). (i) KCh tetramers of 6TM/1P peptides with an intracellular N- and C-terminus. The P region, containing the K^+^-conduction pathway, is situated between the fifth and the sixth TM domain. This group includes the voltage-gated KCh (Kv) and the small and intermediate conductance Ca^2+^-activated KCh (K_Ca_). (ii) Inward rectifier KChs (Kir), the K_ATP_, and the G-protein-coupled KChs. These channels are tetramers formed by four 2TM/1P subunits. The P region is localized between the two TM domains. (iii) KChs assembled by tetramerization of 7TM/1P subunits and, unlike the other groups, the N-terminus is extracellular. In this group, the P region is located between the sixth and seventh TM domain. This group includes the large-conductance members of the K_Ca_ family. Finally, (iv) the K_2P_ family. These channels, formed by dimerization of 4TM/2P proteins, contain two P regions between the first and the second TM domain and between the third and the fourth TM domain [2]. 

KChs regulate many physiological processes, such as cell excitability [3], hormone secretion [4], proliferation [5], and apoptosis [6]. However, the unique expression of the channel is not enough to accomplish such effects. To do so, protein localization within specific membrane compartments or organelles is mandatory. An ever-growing list of examples can be found in the literature, but a few examples are given. Lymphocytes concentrate Kv1.3 at the immune synapse to control Ca^2+^ influx during activation [7] or at the inner mitochondrial membrane to regulate apoptosis [8]. In addition, Kv1.3 targets caveolae in adipocytes participating in the insulin-dependent signaling [9]. In myocytes, Kv11.1 is targeted to the transverse tubular network, Kv7.1 is localized to the peripheral sarcolemma and T-tubules, and, finally, Kv1.5 is concentrated at the intercalated disks and in proximity to Z-lines [10,11]. Finally, neuronal Kv1 channels are targeted to axons to modulate the action potential [12], whereas Kv4 channels are restricted to dendrites where they integrate and propagate excitatory impulses [13].

## 2. Potassium Channel Biogenesis

The nascent channel protein, which contains a signal sequence, is targeted to the endoplasmic reticulum (ER). Although scarce information is available about this step, TM2 functions as a signal sequence in Kv1.3 [14]. The formation of the translocon structure allows for the KCh peptide to enter the ER lumen. Translocation, concomitantly with the integration of the transmembrane segments into the lipid bilayer, defines the membrane topology of the channel. Coordinately, subunit oligomerization takes place. Signatures implicating the tetramerization with members from the same subfamily are located at either the N- or the C-terminus of the subunits. Evidence also implicates transmembrane domains in tetramer formation. Additionally, folding events configure the pore and sensor domains [15]. In addition to structural motifs, the association of auxiliary subunits and accessory proteins is also important in determining protein fate. Some ancillary peptides contain motifs that govern the subcellular location of the functional complex. Protein–protein interactions can provide or hide traffic motifs as well as bend the structure of the KCh, promoting specific targeting. Evidence, therefore, demonstrates that the final destination is not only a one-signal phenomenon but also depends on the equilibrium of several different inputs (motifs, accessory subunits, other partners, etc.). This balance determines the location of a channel for a specific role.

Impaired KCh traffic may lead to severe malfunctions, named channelopathies. Thus, Kv11.1 loss-of-function mutations, causing Long QT syndrome 2 (LQT2), harm channel forward traffic [16,17]. Moreover, Kv11.1 gain-of-function changes, generating short QT syndrome 1 (SQT1), might also cause traffic defects. In the same vein, mutations in Kv11.1 auxiliary subunits, such as KCNE2, disrupt traffic triggering diseases [18,19]. Interestingly, amino acid changes affecting Kv7.1 traffic lead to similar consequences. For instance, deletion of a C-terminal serine of Kv7.1 causes ER retention leading to haploinsufficiency [20]. On the other hand, mutations altering the traffic of K_ATP_ trigger congenital hyperinsulinism and neonatal diabetes mellitus. In this case, many traffic defects are found, all increasing the density of the channel at the membrane. These include disruption of ER forward-trafficking motifs and impairment of endocytosis [21]. Finally, alterations in scaffolding proteins can also dysregulate channel traffic, leading to severe diseases. For instance, mutations in caveolin 3 decrease Kir2.1 membrane density and cause LQT9 syndrome [22].

## 3. Potassium Channel Interactions

### 3.1. Regulatory Subunits

As mentioned above, the functional diversity of KChs depends on the α-subunit conditions and the interaction with auxiliary β-peptides. These β-proteins can regulate both cellular location and function. There are two kinds of regulatory α-subunit: (i) cytosolic peptides, such as KCNAB (Kvβ), K^+^ channel-associated protein (KChAP), and K^+^ channel-interacting proteins (KChIPs); and (ii) small transmembrane proteins (KCNE).

The Kvβ members are soluble proteins that may form up to a 4:4 complex with α-subunits. The association is via the N-terminus of the α-subunit and the C-terminus of the Kvβ. Three genes encode Kvβ subunits in humans: Kvβ1, Kvβ2, and Kvβ3. Sequence alignment shows that Kvβ proteins display a highly conserved C-terminus with more than 80% sequence identity and variable N-terminal sequences. Kvβ subunits are NADPH-dependent aldo-keto reductases. Thus, changes in the oxidoreductase activity may alter the gating of Kv channels. In addition, certain N-termini confer rapid inactivation to otherwise noninactivating Kv channels. Some Kvβ members contribute to the subcellular location of the channel complex [23]. However, the evidence that Kvβ subunits act as chaperones is not convincing. In fact, some Kvβs may either stimulate or attenuate Kv1-channel density at the plasma membrane. Concerning cellular locations, Kvβ subunits may help Kv1 drive axonal compartments. Thus, Kv1.2 is not targeted to the axon unless Kvβ2 is present. This scenario is only possible if Kv1.2 and Kvβ2, after assembly in the ER, travel assembled through the Golgi to reach their final destination [24]. However, work in Kvβ knockout mice argues against a chaperone role because both Kvβ2 knockout and Kvβ1/Kvβ2 double-knockout mice express similar levels of Kv1.1 and Kv1.2 at the neuronal plasma membrane [25]. In summary, although Kvβ subunits may help to target Kv to distinct cellular sites, this is still an open debate.

Members of the KCNE family of K+ channel subunits are short single-transmembrane glycoproteins that exhibit regulatory functions on the kinetics and cellular distribution of the α-subunits of Kv. This family comprises five members (KCNE1–5), and their effect on KCh activity has been widely studied. However, KCNE regulation of Kv traffic is complex. Sometimes, KCNE regulates the channel, whereas, in other situations, the α-subunit drives the KCNE location. For instance, KCNE members associate with Kv7.1. Although KCNEs have limited cell surface expression and lipid raft microdomain targeting, Kv7.1 improves KCNE1 cell surface expression. In addition, upon association with Kv7.1, KCNE1 and KCNE2 relocate partially into lipid rafts. Importantly, complexes situated in these domains contribute to the cardiac IKs (slow component of the delayed rectifier potassium current) [26]. In contrast, KCNE4 and KCNE5 target Kv7.1 out of the rafts [26], and KCNE4 association impairs Kv1.3 membrane expression and caveolar lipid raft localization while retaining the channel in the ER, which fine-tunes channel surface abundance [27].

KChIPs (K^+^ channel interacting proteins), a class of EF-hand calcium-binding proteins, represent another family of auxiliary subunits. Alternative splicing of the four human KChIP genes generates several isoforms (KChIP1–4) with a variant N-terminal region and a conserved C-terminal domain containing four EF hands. KChIPs associate with Kv channels, mostly Kv4, and modulate their cell surface expression as well as their electrophysiological properties. They specifically interact with cytoplasmic domains of Kv4 α-subunits and promote a Ca^2+^-dependent membrane targeting of the channel [28,29,30].

Finally, KChAP (K^+^ channel-associated protein), a member of the activated signal transducer STAT family, binds to the N-terminus of the channels. KChAP raises the functional expression and current amplitude of several Kv, including Kv1.3, Kv2.1, and Kv4.3, in Xenopus oocytes and mammalian cells without altering their biophysical properties, which supports a real chaperone role. Moreover, KChAP and Kvβ regulatory subunits may interact. Thus, Kvβ1.2 counteracts the effects of KChAP on Kv2.1 and Kv4.3, suggesting that the chaperone properties of KChAP could be mediated indirectly by association with Kvβ1.2, which further reveals a complex regulatory scenario for the auxiliary subunits [31,32].

### 3.2. Scaffolding Proteins and Other Partners

KChs may associate with a number of miscellaneous proteins to reach their final destination. Scaffolding proteins help to tether oligomeric complexes, binding multiple members within a signaling pathway in discrete areas of the cell and thus helping the functionality of the Kv complex. Scaffolding proteins contain modular domain structures that recruit many proteins forming complexes. PDZ-domain-containing proteins and SRC homology 3 domains (SH3) are the most notable. Therefore, these peptides connect KChs to the necessary signaling and trafficking molecules driving channels to target organelles. Scaffolding proteins and their partners show highly specific subcellular locations. For example, PSD-95 (postsynaptic density protein 95), also known as SAP-90 (synapse-associated protein 90), localizes in neuronal synaptic regions and drives Kv1.3 to those membrane spots [33]. Discs large homolog 1 (DLG1), also known as synapse-associated protein 97 (SAP97), is also a MAGUK (membrane-associated guanylate kinase) protein known to interact with Kv1.5 and caveolin3 in raft domains [34]. Caveolins are scaffolding proteins that structure caveolar lipid rafts (caveolae), driving Kv into these domains. Direct interaction with the channel occurs via the N-terminal region of caveolin, typically affecting the channel activity [35,36]. Miscellaneous scaffolding proteins are a wide and variable family. Some members, such as CASK (calcium/calmodulin-dependent serine protein kinase) and some MAGUK proteins, are directly involved in driving KChs to their final location. Thus, CASK associates with Kir2 in the brain and heart [37]. Furthermore, other scaffolding proteins, such as AKAP (A-kinase anchoring proteins), anchor channels to the membrane once Kv7.1 reaches the final destination [38,39].

**Figure 2:**
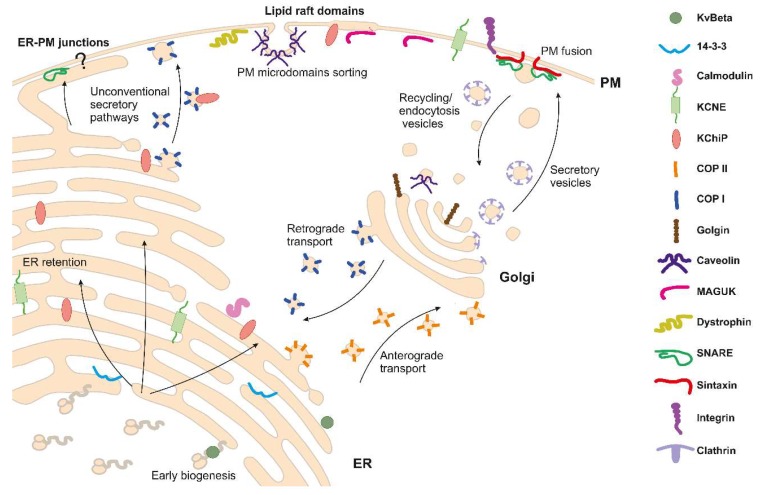
A schematic representation of traffic mechanisms and molecular associations of K^+^ channels through the secretory pathway. Different shapes represent molecules (see list on the right) known to associate with channels in different compartments. Channels are not depicted. Arrows represent major directions of either retrograde or anterograde routes. Newly synthetized peptides, bearing endoplasmic reticulum (ER) signatures, translocate to the rough ER where the association with Kvβ regulatory subunits takes place in early steps of the biogenesis. Different interactions and motifs balance between forward traffic or ER retention. Regulatory subunits, such as KCNEs, and accessory molecules, such K^+^ channel-interacting protein (KChiP), 14-3-3, or calmodulin, interact with channels at this step. Conventional mechanisms dictate that regardless of whether anterograde traffic is promoted, cargo channels are processed along the COPII machinery driving to Golgi. Once in Golgi, channels either forward traffic to the cell surface or undergo retrograde transport to ER via the COPI pathway. Molecules, such as Golgin, caveolin, or clathrin facilitate channel routes to the surface. Accessory molecules, such as MAGUK, dystrophin, syntaxin, integrins, or caveolins stabilize channels at the final destination (e.g., lipid raft microdomains and caveolae represented by a thicker part of the membrane and an invaginated omega-shaped structure, respectively). Turnover mechanisms, such as internalization, are mostly mediated by clathrin-coated pit-dependent endocytosis. Alternatively, unconventional pathways are also facilitated by auxiliary subunits assembling the channels. Thus, export from ER via COPI vesicles requires KChiP interaction. Direct contacts with the plasma membrane (PM) and further stabilization with SNARE at the ER–PM junctions are documented. However, most proteins implicated in this unconventional traffic remain unknown. It is important to highlight that mechanisms could differ depending on the KCh and the interacting protein. See text for further details.

A number of proteins, not included in any specific regulatory group, are widely involved in Kv channel trafficking, and regulation will also be mentioned in this review. Thus, integrins, glycoproteins involved in adhesion to the extracellular matrix, regulate channels, such as Kv1.3. Calmodulin (CaM), a calcium-binding protein that regulates several cell processes in response to Ca^2+^, governs the traffic of Kv7 channels. Vesicle proteins, such as syntaxins and synaptotagmins, are related to movements of neuronal channels along the trafficking pathway, while modulating the adhesion or fusion to the membrane or other organelles [34]. Regulatory molecules, such as 14-3-3, will also be mentioned in this work. Figure 2 represents a cartoon compiling the most representative KCh auxiliary subunits and scaffolding proteins affecting traffic throughout the vesicular pathway.

## 4. Subcellular Targeting of Potassium Channels

### 4.1. Endoplasmic Reticulum

After translation, KChs are located in the ER exit sites (ERES) and are packed into COPII-coated vesicles and transported via microtubules to the ER-Golgi intermediate compartment (ERGIC). Many motifs are essential for such forward trafficking. For instance, diacidic signatures at the C-terminus of Kir channels are abundant. Thus, there is a VLSEVDETD motif in Kir1 channels, FYCENE in Kir2.1 channels [40], ELETEEEE in Kir3.2A channels, NQDMEIGV in Kir3.4 channels, and DXE in Kir6.2 channels [21]. Similar clusters are also present in other KChs, such as K_2P_3.2 (EDE) [41], Kv1.3 (YMVIEE) [42], and the HRETE sequence of Kv1 channels, which exert great influence in trafficking. Furthermore, an acidic extension of this HRETE in the C-terminus of the mouse Kv1.3 (ETEGE) [43] and a cluster of acidic residues (DDXXDXXX) in a splice variant of K_Ca_1.1 [44] have also been implicated.

Another example is the VXXSL sequence, which is essential for efficient Kv1.4 anterograde traffic. Variations of this domain with other members in the same family explain the relative surface targeting efficiencies for each channel. Swapping such a domain between channels interchanges their forward trafficking efficiencies [45]. Finally, mutations at the C-terminal cyclic nucleotide-binding domain of Kv11.1 can also cause defective anterograde trafficking of the channel with fatal consequences [46].

The association of Kvβ subunits also has a notable effect on the ER exit of Kvs. The interaction between Kv1 α and Kvβ subunits occurs in the ER early in channel biosynthesis. This assembly sometimes increases the traffic of channels to the plasma membrane. For example, Kvβ2 and Kv1.2 associate early in channel biogenesis, interacting cotranslationally, even before Kv1.2 is completely translated and translocated into the ER. The β-subunit also affects the *N*-glycosylation of the channel, mediating proper folding and traffic throughout the ER journey [24].

In addition to forward trafficking motifs, ER quality controls prevent the anterograde traffic of misfolded channels. Defective proteins may undergo several rounds of folding before irreversibly being targeted for ER-associated degradation (ERAD). This mechanism mediates the retrotranslocation of the protein back to the cytosol and a subsequent ubiquitin-dependent proteasomal degradation. The mechanism for ER retention mostly consists of the exposure of retrieval motifs. Thus, di- or tri-basic signatures, recognized by COPI vesicle coats, retrieve the defective channel from Golgi back to the ER [47]. An RXR motif located at the C-terminus of K_ATP_ channels is an example [48]. Although many KChs also contain RXR domains, only in Kv11.1 (ERG) [49] have the domains been definitively confirmed. A similar basic C-terminal signal (KRR) in K_2P_3.2 binds to COPI and drives the channel back to the ER [50]. Other ER retention signals might be less discrete but otherwise widespread. For instance, hydrophobic sequences situated at the N-terminus of Kv4.2 promote channel aggregation, misfolding, and subsequent degradation [29].

ER retention can be physiological and not due to unsatisfactory quality control. For instance, pore-based retention mechanisms have been described for Kv1 and Kv7. The pore region of Kv1.1 contains two critical residues for the ER retention of this channel [51]. Similarly, a pore residue in Kv7.3 is responsible for an inefficient forward trafficking [52]. Finally, a splice variant of K_Ca_1.1 exhibits another retention/retrieval ER mechanism, which implies a hydrophobic domain (CVLF) in the intracellular S0–S1 loop [53].

Many ancillary proteins may alter KCh surface targeting. Thus, KCNE subunits are also key regulators of Kv traffic in the immune system. As mentioned above, KCNE4 impairs Kv1.3 membrane targeting while retaining the channel in the ER by two mechanisms [27]. First, KCNE4 masks the forward trafficking motif (YMVIEE) at the C-terminus of Kv1.3, and second, KCNE4 contains a potent ER retention motif that further limits the surface expression of the complex [54]. KChIPs are also involved in both membrane targeting and retention of KChs. Coexpression of KChIPs1–3 causes a dramatic redistribution of Kv4.2, releasing intrinsic ER retention and improving the cell surface expression via a mechanism that may involve masking of a cytoplasmic trafficking and/or solubility determinant in excitable cells [29]. In contrast, KChIP2 promotes ER retention when coexpressed with Kv1.5 [55]. On the other hand, Kv7 channels, such as Kv7.2, require CaM to properly fold and adopt an active conformation to exit the ER. Thus, CaM acting as a Ca^2+^ sensor confers Ca^2+^-dependence for the cell surface trafficking of the channel [56,57]. Similarly, the Kv7.2–Kv7.3 complex requires a Ca^2+^/CaM-dependent mechanism for targeting the axonal surface in neuronal cells [58,59]. In this vein, K_Ca_ channels are monomers at this step of the anterograde traffic. C-termini of the monomers interact constitutively with CaM, which allows for the multimerization and thus promotes the surface expression of the functional channel [60,61]. Finally, K_ATP_ functional channels are formed by the association of Kir6 subunits and SUR proteins stabilized in macromolecular dynamic complexes that contain kinases, ankyrins, and other cytoskeleton and adaptor proteins. Both SUR and Kir6 contain putative ER retention motifs (RXR) that retain every subunit in the ER when expressed alone. Their association masks ER retention motifs, improving the membrane targeting of the functional complex [48,62].

### 4.2. Golgi Sorting

The Golgi apparatus is at the center of the secretory pathway. The Golgi’s structure of stacked flattened cisternae confers the proper environment for accurate protein maturation. Proteins entering the ERGIC are transferred through the *cis*-, *medial*-, and *trans*-cisternae to be further processed. The *trans*-Golgi network (TGN) organizes the sorting of proteins to the plasma membrane, where cargo proteins concentrate into specific pools of nascent clathrin-coated vesicles. The sorting signals that regulate such stages of KCh maturation remain largely unexplored. *Trans*-Golgi export of Kir channels is mediated by a cluster of residues located within the confluence of the cytoplasmic N- and C-terminal domains. This creates a tertiary structure that contributes to the interaction with adaptor protein complex 1 (AP1), driving the incorporation of Kir channels into clathrin-coated vesicles. This domain includes basic residues at the N-terminus and a hydrophobic cleft at the C-terminus [63]. Furthermore, the sorting of KChs to specific plasma membrane compartments is already defined in the TGN. For instance, neuronal Kv2.1 and Kv4.2 are sorted into distinct pools of transport vesicles specifically targeted to proximal (Kv2.1) or distal (Kv4.2) dendrite compartments. Kv2.1 vesicles traffic by a mechanism involving myosin and actin filaments, whereas Kv4.2 vesicles depend on kinesin and microtubules [64]. Several mutations that impair the post-Golgi sorting process cause the mislocalization of these channels in the neuronal membranes. Kv2.1 localizes in large surface clusters in soma and proximal dendrites due to a serine-enriched C-terminal motif, known as the proximal restriction and clustering (PRC) signal [65]. Disruption of this motif triggers incorrect post-Golgi sorting, causing aberrant localization of Kv2.1 to distal dendrites. Similarly, the C-terminal dileucine motif of Kv4.2 drives dendritic targeting [66], and mutations cause impaired vesicular sorting of the channel [64].

In addition to polarized traffic, the packing of KChs into specialized membrane microdomains occurs at the TGN. For instance, Kv1.3 lipid raft targeting depends on a caveolin interaction through its N-terminal caveolin-binding domain (QRQVWLLF). Such an interaction could be functional at the TGN, where lipid rafts begin to structure as cholesterol is concentrated [67]. Altogether, the TGN can not only direct the journey of KCh nascent vesicles but also the channel affinity for structured plasma membrane domains.

Anterograde traffic of several proteins through the Golgi requires specific golgin molecules. Thus, Kir1.1, Kv1.5, and Kv4.3 require Golgin-160 to reach the plasma membrane, even though there is no obvious sequence similarity in the cytoplasmic tails of these channels. Kir1.1 membrane traffic is also promoted by GM130. In contrast, GM130 is responsible for the Kv11.1 retention at this traffic step [68]. Furthermore, Kir2.1 is recognized by Golgin-97 as an export cargo to be delivered into the TGN [69]. Consequently, some Kir2.1 N-terminal motifs are required to exit the Golgi as it reaches the membrane [70].

### 4.3. Plasma Membrane Arrangement

Eventually, KChs are inserted into the plasma membrane. Mechanisms to maintain KChs at their target destination are needed in polarized cells. A major mechanism for protein retention involves KCh anchoring to the cytoskeleton. For instance, neuronal Kv7 channels are retained at the axonal initial segment by being anchored to the actin cytoskeleton through a C-terminal ankyrin-G-binding motif (I/LAXGES/TDXE/D) [71,72]. Likewise, cortactin immobilizes Kv1.3 through an SH3 C-terminal domain (PQTP). Such an interaction at the immunological synapse suggests that the polarization of the T cell would promote Kv1.3 trapping at active sites of the actin network [73]. Other arrangement mechanisms include coclustering by protein–protein interactions. For instance, a PDZ-binding motif at the C-terminal end of Kv1 channels (S/TXV) facilitates Kv1 immobilization by PSD-95 interaction [74]. The effects of protein–lipid interactions on the membrane organization of KChs remain elusive, although the effects are expected to be considerable given the number of KChs that target lipid raft microdomains [75,76].

Kvβ subunits modulate the cell surface expression of many Kv1 channels. Kvβ members not only promote Kv1.2 membrane targeting but also need the NADP+-binding pocket to do so. Defects in the cofactor-binding pocket reduce axonal targeting of the channel in hippocampal neurons [77]. In arterial myocytes, Kvβ2, taking a chaperone role, facilitates Kv1.5 trafficking and membrane localization [78].

SNARE proteins, syntaxins, and integrins help channels to reach the membrane. SNARE proteins are membrane-integrated proteins responsible for vesicle-associated exocytosis, but they can also act as a recruitment signal for proteins that must reach the membrane. KAT1, a plant Shaker-related member, shares a number of structural features with the voltage-sensitive Kv superfamily of eukaryotes. KAT1 binds the plasma membrane protein SYP121, a Q-SNARE protein, through a conserved RYXXWE motif located at the cytosolic surface of the voltage-sensor domain [79]. K_2P_ and Kv2.1, as well as many other Kvs, interact with SNARE proteins and syntaxins, shaping the intracellular itinerary of the channels. However, the mechanisms by which SNARE proteins affect vesicular transport are unknown [80,81].

Scaffolding proteins are also very important for membrane trafficking and protein stabilization at specific locations. The C-terminal PDZ domains of Kir channels interact with several partners that regulate intracellular trafficking. Members from the MAGUK family, including SAP91, PSD-95, Chapsyn-110, SAP102, and Veli or actin-binding LIM proteins, are scaffolding proteins related to the membrane expression of Kir channels. Thus, PSD-95 facilitates Kir2.3 clustering on the plasma membrane [82], and SAP97 regulates Kir2 traffic [83]. In addition, dystrophin-associated complexes are also involved in the anchoring and stabilization of these channels at the plasma membrane [84].

In addition to the conventional secretory pathway, which requires COPII vesicles to exit the ER and reach the membrane, some proteins use unconventional routes to target the cell surface. These nonclassical pathways are complex and usually comprise cargo molecules with an unknown signature [85,86]. For instance, Kv4.2 uses COPI vesicles, instead of COPII, but only in coexpression with KChIP1 [30]. The CFTR (cystic fibrosis transmembrane conductance regulator) chloride channel uses the ER–plasma membrane (PM) junctions, which bypass the Golgi apparatus to reach the membrane [87]. Concerning KChs, Kv2.1 targets and stabilizes these domains. In fact, Kv2.1 provides platforms for delivery and retrieval of multiple membrane proteins [88,89]. The Golgi bypass has recently been under analysis. Thus, unknown combinations of SNARE proteins and/or specific molecules yet to be identified would drive KChs to the membrane unconventionally.

Spatially, KChs can be located in specific membrane microdomains, such as lipid rafts. Raft domains are membrane fractions enriched in sphingolipids and cholesterol that serve as scaffolding regions where signal transduction pathways interface. Proteins reach these domains via different mechanisms, such as lateral diffusion, the formation of lipid “shells” surrounding the channel, which increase the affinity for rafts, protein–lipid interactions, such as palmitoylation (see below), or protein–protein interactions [75]. In this last scenario, caveolin acts as a scaffolding protein driving ion channels to raft domains. For instance, an N-terminal motif for the caveolin-1 interaction (FQRQVWLL) has been described in Kv1.3 [67]. Caveolins, by impairing lateral diffusion, increase the time that KChs are located in these platforms. In addition, KChIP proteins also govern Kv4.3 targeting to lipid raft domains [90]. Finally, PSD-95 is partially responsible for the lipid raft targeting of Kv1.4 and protects Kv1.3 against phorbol 12-myristate 13-acetate (PMA)-induced ubiquitination and endocytosis [33,91]. Kv2.1, Kv1.5, or Kir3.1 are also found in these domains [75], but no direct interaction with scaffolding or auxiliary proteins has been reported.

Mechanisms of membrane arrangement also involve channel internalization. Lysosomal endocytosis is not always associated with degradation. For instance, Kir6.x target to the plasma membrane when associated with SUR subunits. However, differential interactions between SUR members and Kir6.x channels condition the subcellular location of the complex. SUR1 targets Kir6 to the plasma membrane, while Kir6/SUR2 localizes mainly in endosomal and lysosomal compartments. In cardiomyocytes, Kir6/SUR2 complexes in endosomal compartments are in constant recycling from the membrane to endosomes. Thus, these oligomers represent a pool of available channels to dynamically regulate heart function [92]. Similarly, K_Ca_2.3, a long-life membrane protein, undergoes dynamin- and Rab5-dependent endocytosis that generates a recycling endosome compartment for a quick and dynamic K_Ca_2.3 return to the cell surface. This mechanism is responsible for K_Ca_2.3 long-life membrane expression [61,93].

## 5. Post-Translational Modifications Regulating Traffic

### 5.1. N-Glycosylation

*N*-glycosylation promotes the surface expression of many KChs probably by reducing protein dynamics and thus increasing stability. *N*-glycosylation adds oligosaccharide chains on the luminal N residue of the N-X-S/T/C consensus sequence (where X can be any amino acid except P). This process starts in the ER, where a preassembled polymannose oligosaccharide is cotranslationally transferred to the N residue. Once in Golgi, sequential enzymatic reactions increase the complexity of the initially added glycan tree [94]. *N*-glycosylation at the S1–S2 loop favors the surface expression of some Kv1 channels, such as Kv1.4 [95], Kv1.2 [96], and Kv1.3 [97]. With similar consequences, Kv12.2 and Kv10.1 are glycosylated at the S5–P loop [98,99] and K_2P_3.1 at the first loop [100]. More controversial is the effect of *N*-glycosylation on the Kv11.1 traffic [101,102]. In fact, *N*-glycosylation regulates gating rather than the surface expression of Kv1.1 [95]. Interestingly, although Kv3.1 *N*-glycosylation is not essential for plasma membrane targeting [103], *N*-glycan structures guide the spatial distribution of the channel once at the plasma membrane, with major effects on cell adhesion, migration, and cell–cell interactions [104,105].

*N*-glycosylation of regulatory subunits or scaffolding proteins can also modify the traffic of KChs. For instance, glycosylation of some β regulatory proteins, such as KCNE1, having one or two N-glycan trees, drives the biogenic process of the Kv7.1–KCNE1 complex. KCNE1 can also be O-glycosylated, and altered isoforms have deleterious effects on the biogenesis of the peptide as well as on the Kv7.1–KCNE1 complex [106].

### 5.2. Phosphorylation

Phosphorylation is a reversible and dynamic PTM consisting of the transfer of the γ-phosphate group of ATP to the hydroxyl group on the side chains of Ser, Thr, or Tyr residues. Usually, the addition of the phosphate group causes significant changes in protein conformation, inducing new protein–protein interactions. Phosphorylation is enzymatically catalyzed by protein kinases and reversed by phosphatases. The existence of thousands of phosphosites and hundreds of protein kinases and phosphatases makes phosphorylation a highly versatile PTM with many different implications in the activity and, to our concern, the trafficking and partitioning of KChs.

For instance, phosphorylation of an N-terminal Ser in Kir1.1 promotes the export of the channel, overriding ER-retention signals [107]. In contrast, ERK1/2-dependent phosphorylation of C-terminal T495 of Kv1.3 mediates the EGF-dependent endocytosis of the channel [108]. Similarly, protein kinase A (PKA)-induced phosphorylation of S552 of Kv4.2 causes internalization [109]. Intriguingly, the same S552 requires phosphorylation for an efficient KChIP-dependent Kv4.2 surface targeting [110]. This fact supports the hypothesis that phosphorylation of the same residue may trigger opposing effects depending on the life-stage of the protein. Another example of the versatility of phosphorylation is that Kv1.2. phosphorylation at a C-terminal Ser in Kv1.2 enhances the cell surface expression of the channel [111], whereas phosphorylation of an N-terminal Tyr promotes rapid internalization [112]. Finally, phosphorylation not only regulates both forward trafficking and turnover of KChs but also can determine their arrangement within the membrane, e.g., dephosphorylation of a large Ser/Thr-rich domain in Kv2.1. The C-terminal tail dramatically disperses the somatodendritic clusters of the channel uniformly throughout the membrane of the neuron [113].

14-3-3 is a ubiquitous protein that acquires scaffolding roles. This protein participates in the intracellular trafficking of membrane proteins by recognizing phosphoserine-containing motifs in the target. 14-3-3 participates in the surface expression of K_2P_ channels, such as K_2P_3.1 or K_2P_9.1. K_2P_5.1 associates with 14-3-3 at the early stages of biogenesis in the ER. This association facilitates the anterograde traffic of the channel to reach the plasma membrane [114].

### 5.3. Redox Modifications

The formation of reactive oxygen and nitrogen species (ROS/RNS) can induce the post-translational oxidative modification of proteins, usually affecting Cys residues due to their highly reactive thiol group. Redox mechanisms affecting KCh trafficking remain highly unexplored and yet are very promising, especially for our understanding of the progression of many pathologies, such as cancer or cardiovascular diseases [115]. For instance, oxidative stress reduces surface expression of cardiac Kv1.5 via sulfenic acid modification of a C-terminal Cys [116]. Likewise, hypoxia disrupts Kv1.3 clathrin-mediated forward trafficking from *trans*-Golgi to the plasma membrane in T-lymphocytes [117].

### 5.4. Lipidation

Protein lipidation is an emerging field and regulates different properties of ion channel function. Lipidation increases protein hydrophobicity. Therefore, this process may regulate the affinity of membrane proteins for specialized membrane microdomains.

*S*-acylation is the attachment of a fatty acid to an intracellular Cys via a thioester linkage. Unlike other lipidations, *S*-acylation is reversible due to the lability of the thioester bond. Therefore, proteins can undergo several rounds of acylation/deacylation during their lifetime, leading to multiple regulatory scenarios. In most cases, the identity of the acyl chain attached to the protein is unknown. However, palmitoylation modulates the trafficking of some KChs. Palmitoylation of a single Cys residue at the C-terminus of Kv1.5 decreases the surface expression of the channel, probably raising the rate of internalization [118]. The large-conductance K_Ca_1.1 is palmitoylated by different palmitoyl acyltransferases at different residues. Palmitoylation of the S0–S1 Cys enhances the surface expression of the channel. Interestingly, palmitoylation also regulates ER exit, but depalmitoylation of K_Ca_1.1 by APT1 largely retains the channel at the TGN, suggesting a major checkpoint for forward trafficking at Golgi exit [119,120]. Palmitoylation of PSD-95 increases Kv1.4 targeting to lipid rafts. However, when manipulating PSD-95, no apparent modifications in Kv4.2 membrane targeting are observed. These results support the hypothesis that different subpopulations of lipid rafts may require specific signals or ancillary associations [91]. Similarly, evidence supports the hypothesis that KChIP participates in lipid raft targeting only when palmitoylated [90].

Myristoylation also affects the traffic of KChs. *N*-Myristoylation is the attachment of a myristoyl fatty acid to the N-terminal Gly of a target protein, usually cotranslationally via an amide bond. Similar to other protein lipidations, myristoylation can regulate protein affinity for specific membrane localizations while controlling protein–protein and protein–lipid interactions. Oddly, myristoylation also occurs post-translationally on internal residues via ester or amide bonds. K_Ca_1.1 undergoes myristoylation on Ser and Thr residues of intracellular loops 1 and 3. This lipidation modulates K_Ca_1.1 traffic to the plasma membrane [121].

### 5.5. Ubiquitination

Ubiquitination is the covalent attachment of a ubiquitin to a Lys residue of a target protein. Strikingly, ubiquitin itself can be ubiquitinated at any Lys, which increases the complexity and regulatory potential of this PTM. Monoubiquitination mostly triggers endocytosis and lysosomal degradation. For instance, Kir1.1 is endocytosed in response to monoubiquitination at the N-terminus [122]. In contrast, polyubiquitination targets Kv11.1 to proteasomal degradation [123]. Nedd4-2 is a ubiquitin ligase that targets KChs. Thus, Kv7.1 undergoes ubiquitin-dependent degradation via interaction with Nedd4-2 at the C-terminal PY motif of the channel (PPXY) [124]. However, Kv1.3, with no apparent PY motif, undergoes Nedd4-2-mediated ubiquitin-dependent endocytosis [33].

## 6. Mechanisms for Organelle Targeting

### 6.1. Lysosomes

Two types of KChs have been identified in lysosomes: TMEM175, which is a novel KCh with a noncanonical structure that regulates lysosomal membrane potential and pH, and large conductance, Ca^2+^-activated K^+^ (BK) channels, which regulate lysosomal Ca^2+^ movements. While the lysosomal targeting mechanisms of TMEM175 are unknown, dileucine motifs at the C-terminus of the BK channels have been identified. Dysfunction of lysosomal KChs is related to a variety of neurodegenerative disorders [125,126].

### 6.2. Nucleus

Some KChs have been identified at the nuclear envelope of different cell types. A putative role has been postulated, suggesting that KChs control nuclear ΔΨ, nucleoplasmic calcium movements, and, ultimately, gene expression. The targeting mechanisms and specific localization within the nuclear envelope are unclear, as is the case for K_ATP_ [127], Kir2.2 [128], K_Ca_3.1 [129], BK [130], and Kv1.3 [131].

The outer nuclear membrane (ONM) is an extension of the ER. Nevertheless, it contains a distinct protein composition; hence, the existence of specific targeting mechanisms from the ER is evident. The inner nuclear membrane (INM) connects the ONM through the lateral channels of the nuclear pore complex (NPC). Integral proteins may travel from the ONM across such lateral channels to locate finally at the INM. Channels can do so by free lateral diffusion or accompanied by importins, which can recognize nuclear localization sequences (NLS) within the cytosolic domains of transmembrane proteins. Once at the INM, proteins can be retained by interactions with chromatin and/or laminins [132]. NLS can be monopartite (a short basic sequence) or bipartite (two short basic sequences separated by a linker). Although the oncogenic potassium channel Kv10.1 contains a bipartite C-terminal NLS, its removal does not affect the INM localization. Interestingly, a mechanism by which plasma membrane Kv10.1 and not ER-localized Kv10.1 relocate to the INM has been postulated [133].

### 6.3. Mitochondria

An increasing number of KChs are reported in the inner mitochondrial membrane (IMM). Although the physiological and pathophysiological role of mitochondrial channels is not completely understood, intense work has been performed that points to a major role in the regulation of energy metabolism [134], apoptosis [135], and autophagy [136]. However, little is known about the mechanisms governing the mitochondrial targeting of KChs. The inclusion of a splice exon at the C-terminal end of K_Ca_1.1 specifically sorts the channel into mitochondria [137]. Similarly, a short isoform of Kir1.1 contains a canonical N-terminal mitochondrial targeting motif, which drives the channel to the IMM [138]. For the rest of mitochondrial KChs, the sorting mechanisms can only be speculated. To date, the list includes K_Ca_3.1 [139], K_Ca_2.2 [140], Kv7.4 [141], K_2P_3.2 [142], K_Na_1.2 [143], and Kv1.3 [8].

Mitochondrial KChs are encoded by the nuclear genome because no obvious gene for a KCh is found in the mitochondrial genome. The channel protein is synthetized by cytosolic ribosomes and likely translocated into mitochondria through translocase of the outer membrane (TOM)/translocase of the inner membrane (TIM) system. Although the abovementioned Kir1.1 presents a mitochondrial targeting sequence at the N-terminus, this is far from a general mechanism, as demonstrated by BK channels. In the case of Kv1.3, an even more complex scenario is hypothesized. Thus, the plasma membrane channel could be transferred to mitochondria via ER–mitochondria contact sites, the so-called mitochondria-associated membranes (MAM) structures [6]. This idea is reinforced by the fact that *N*-glycosylated proteins can be transported from ER to chloroplast [144] and by the discovery of direct transfer of viral proteins between ER and mitochondria [145]. However, a very interesting study about the mitochondrial sorting of a viral KCh supports the view that the channel is translocated into mitochondria through the canonical TIM/TOM system, guided by structural information encoded by the C-terminus of the channel [146].

## 7. Heterotetramerization

In addition to the influence of ancillary molecules on KCh traffic, heterotetrameric associations, mostly with other members of the same family, cause potential rerouting. Kv1.3 associates with Kv1.1, Kv1.2, and Kv1.4 in the brain, and Kv1.3/Kv1.5 hybrids are found in macrophages. While homomeric Kv1.5 does not target lipid raft domains, heterotetrameric channels with Kv1.3 redirect the Kv1.5 to caveolar domains. Moreover, different stoichiometry within the complex shows different cell distribution and electrophysiological properties [147]. Furthermore, heteromeric assembly of neuronal channels generates variable phenotypes. Kv1.1 is mainly localized in the ER, Kv1.4 at the plasma membrane, and Kv1.2 is found in both locations. Kv1.1. and 1.2 increase their membrane expression when assembled with Kv1.4 in a dose-dependent manner. However, when Kv1.1 is saturated, the complex formed with Kv1.4 reduces its surface targeting significantly. This again suggests an effect of the α-subunit stoichiometry on the targeting of the complexes [148,149].

Kv7.2/Kv7.3 generates the M-current, which controls neuronal excitability. Kv7.3 does not form functional homotetramers on the membrane but needs the association of Kv7.2 to escape the ER and reach the cell surface [52]. Furthermore, functional Kv7.2/Kv7.3 heterotetramers associate with ankyrin-G targeting the axon initial segment [72].

Furthermore, complex hetero-oligomerization can also happen between different ion channels. For instance, Kir2.1 and Nav1.5 form macromolecular complexes that participate in cardiac excitability by interacting with several other partners at early stages of their secretory pathway. The complex preassembles during early forward traffic and exits the Golgi apparatus by AP1 recognition. Moreover, traffic alterations of one member affect the whole complex secretion. Thus, retention of Kir2.1 at the Golgi impairs Nav1.5 membrane targeting [150,151].

## 8. Concluding Remarks

The channel complex contains several motifs that drive its journey throughout the vesicular pathway (Figure 2). Cooperatively, association with auxiliary subunits and other interacting partners helps the channel to reach the final destination. These associations can mask or expose channel motifs that contain traffic signals. A heteromeric composition of the channel protein can also modulate traffic. Additionally, channels can undergo several PTMs with direct effects on their traffic. Mechanisms of forward traffic will favor ER exit, driving the channel to further quality controls. KChs can be either retained or retrieved into the ER. Once in the Golgi, the channel will be further matured and sorted within specific vesicles. At the plasma membrane, the channel distribution can be further spatially organized into specific microdomains. Evidence supports unconventional pathways, including direct arrival to the plasma membrane by bypassing the Golgi. Specific mechanisms can also drive the channel to other destinations within the vesicular pathway, such as lysosomes or the nuclear membrane. Additionally, KChs can reach organelles outside the anterograde vesicular pathway by alternative mechanisms, such as mitochondrial targeting. The number of channels at the destination is of great relevance for cellular physiology. Thus, a fine regulation of turnover is crucial.

Considering this, traffic regulation shows a large number of possibilities for accomplishing the proper function of channels. A certain variable in the whole process can trigger differential effects, not only at the cellular level but also at the level of whole-body homeostasis.

## References

[1] MacKinnon R. (2003). Potassium channels. FEBS Lett..

[2] Gutman G.A., Chandy K.G., Adelman J.P., Aiyar J., Bayliss D.A., Clapham D.E., Covarriubias M., Desir G.V., Furuichi K., Ganetzky B. (2003). International Union of Pharmacology. XLI. Compendium of voltage-gated ion channels: Potassium channels. Pharmacol. Rev..

[3] Jeevaratnam K., Chadda K.R., Huang C.L., Camm A.J. (2018). Cardiac Potassium Channels: Physiological Insights for Targeted Therapy. J. Cardiovasc. Pharmacol. Ther..

[4] Ashcroft F.M., Rorsman P. (2013). K(ATP) channels and islet hormone secretion: New insights and controversies. Nat. Rev. Endocrinol..

[5] Comes N., Serrano-Albarras A., Capera J., Serrano-Novillo C., Condom E., Ramon Y.C.S., Ferreres J.C., Felipe A. (2015). Involvement of potassium channels in the progression of cancer to a more malignant phenotype. Biochim. Biophys. Acta.

[6] Szabo I., Zoratti M., Gulbins E. (2010). Contribution of voltage-gated potassium channels to the regulation of apoptosis. FEBS Lett..

[7] Panyi G., Vamosi G., Bacso Z., Bagdany M., Bodnar A., Varga Z., Gaspar R., Matyus L., Damjanovich S. (2004). Kv1.3 potassium channels are localized in the immunological synapse formed between cytotoxic and target cells. Proc. Natl. Acad. Sci. USA.

[8] Szabo I., Bock J., Jekle A., Soddemann M., Adams C., Lang F., Zoratti M., Gulbins E. (2005). A novel potassium channel in lymphocyte mitochondria. J. Biol. Chem..

[9] Perez-Verdaguer M., Capera J., Ortego-Dominguez M., Bielanska J., Comes N., Montoro R.J., Camps M., Felipe A. (2018). Caveolar targeting links Kv1.3 with the insulin-dependent adipocyte physiology. Cell. Mol. Life Sci..

[10] Rasmussen H.B., Moller M., Knaus H.G., Jensen B.S., Olesen S.P., Jorgensen N.K. (2004). Subcellular localization of the delayed rectifier K(+) channels KCNQ1 and ERG1 in the rat heart. Am. J. Physiol. Heart Circ. Physiol..

[11] Eldstrom J., Van Wagoner D.R., Moore E.D., Fedida D. (2006). Localization of Kv1.5 channels in rat and canine myocyte sarcolemma. FEBS Lett..

[12] Bean B.P. (2007). The action potential in mammalian central neurons. Nat. Rev. Neurosci..

[13] Johnston D., Christie B.R., Frick A., Gray R., Hoffman D.A., Schexnayder L.K., Watanabe S., Yuan L.L. (2003). Active dendrites, potassium channels and synaptic plasticity. Philos. Trans. R. Soc. Lond. Ser. B Biol. Sci..

[14] Tu L., Wang J., Helm A., Skach W.R., Deutsch C. (2000). Transmembrane biogenesis of Kv1.3. Biochemistry.

[15] Deutsch C. (2002). Potassium channel ontogeny. Annu. Rev. Physiol..

[16] Anderson C.L., Delisle B.P., Anson B.D., Kilby J.A., Will M.L., Tester D.J., Gong Q., Zhou Z., Ackerman M.J., January C.T. (2006). Most LQT2 mutations reduce Kv11.1 (hERG) current by a class 2 (trafficking-deficient) mechanism. Circulation.

[17] Furutani M., Trudeau M.C., Hagiwara N., Seki A., Gong Q., Zhou Z., Imamura S., Nagashima H., Kasanuki H., Takao A. (1999). Novel mechanism associated with an inherited cardiac arrhythmia: Defective protein trafficking by the mutant HERG (G601S) potassium channel. Circulation.

[18] Yang Y., Xia M., Jin Q., Bendahhou S., Shi J., Chen Y., Liang B., Lin J., Liu Y., Liu B. (2004). Identification of a KCNE2 gain-of-function mutation in patients with familial atrial fibrillation. Am. J. Hum. Genet..

[19] Humphries E.S., Dart C. (2015). Neuronal and Cardiovascular Potassium Channels as Therapeutic Drug Targets: Promise and Pitfalls. J. Biomol. Screen..

[20] Gouas L., Bellocq C., Berthet M., Potet F., Demolombe S., Forhan A., Lescasse R., Simon F., Balkau B., Denjoy I. (2004). New KCNQ1 mutations leading to haploinsufficiency in a general population; Defective trafficking of a KvLQT1 mutant. Cardiovasc. Res..

[21] Sivaprasadarao A., Taneja T.K., Mankouri J., Smith A.J. (2007). Trafficking of ATP-sensitive potassium channels in health and disease. Biochem. Soc. Transact..

[22] Vaidyanathan R., Vega A.L., Song C., Zhou Q., Tan B.H., Berger S., Makielski J.C., Eckhardt L.L. (2013). The interaction of caveolin 3 protein with the potassium inward rectifier channel Kir2.1: Physiology and pathology related to long qt syndrome 9 (LQT9). J. Biol. Chem..

[23] Martens J.R., Kwak Y.G., Tamkun M.M. (1999). Modulation of Kv channel alpha/beta subunit interactions. Trends Cardiovasc. Med..

[24] Shi G., Nakahira K., Hammond S., Rhodes K.J., Schechter L.E., Trimmer J.S. (1996). Beta subunits promote K+ channel surface expression through effects early in biosynthesis. Neuron.

[25] McCormack K., Connor J.X., Zhou L., Ho L.L., Ganetzky B., Chiu S.Y., Messing A. (2002). Genetic analysis of the mammalian K+ channel beta subunit Kvbeta 2 (Kcnab2). J. Biol. Chem..

[26] Roura-Ferrer M., Sole L., Oliveras A., Dahan R., Bielanska J., Villarroel A., Comes N., Felipe A. (2010). Impact of KCNE subunits on KCNQ1 (Kv7.1) channel membrane surface targeting. J. Cell. Physiol..

[27] Sole L., Roura-Ferrer M., Perez-Verdaguer M., Oliveras A., Calvo M., Fernandez-Fernandez J.M., Felipe A. (2009). KCNE4 suppresses Kv1.3 currents by modulating trafficking, surface expression and channel gating. J. Cell Sci..

[28] Flowerdew S.E., Burgoyne R.D. (2009). A VAMP7/Vti1a SNARE complex distinguishes a non-conventional traffic route to the cell surface used by KChIP1 and Kv4 potassium channels. Biochem. J..

[29] Shibata R., Misonou H., Campomanes C.R., Anderson A.E., Schrader L.A., Doliveira L.C., Carroll K.I., Sweatt J.D., Rhodes K.J., Trimmer J.S. (2003). A fundamental role for KChIPs in determining the molecular properties and trafficking of Kv4.2 potassium channels. J. Biol. Chem..

[30] Hasdemir B., Fitzgerald D.J., Prior I.A., Tepikin A.V., Burgoyne R.D. (2005). Traffic of Kv4 K+ channels mediated by KChIP1 is via a novel post-ER vesicular pathway. J. Cell Biol..

[31] Kuryshev Y.A., Gudz T.I., Brown A.M., Wible B.A. (2000). KChAP as a chaperone for specific K(+) channels. Am. J. Physiol. Cell Physiol..

[32] Kuryshev Y.A., Wible B.A., Gudz T.I., Ramirez A.N., Brown A.M. (2001). KChAP/Kvbeta1.2 interactions and their effects on cardiac Kv channel expression. Am. J. Physiol. Cell Physiol..

[33] Martinez-Marmol R., Styrczewska K., Perez-Verdaguer M., Vallejo-Gracia A., Comes N., Sorkin A., Felipe A. (2017). Ubiquitination mediates Kv1.3 endocytosis as a mechanism for protein kinase C-dependent modulation. Sci. Rep..

[34] Folco E.J., Liu G.X., Koren G. (2004). Caveolin-3 and SAP97 form a scaffolding protein complex that regulates the voltage-gated potassium channel Kv1.5. Am. J. Physiol. Heart Circ. Physiol..

[35] Davies L.M., Purves G.I., Barrett-Jolley R., Dart C. (2010). Interaction with caveolin-1 modulates vascular ATP-sensitive potassium (KATP) channel activity. J. Physiol..

[36] Couet J., Li S., Okamoto T., Ikezu T., Lisanti M.P. (1997). Identification of peptide and protein ligands for the caveolin-scaffolding domain. Implications for the interaction of caveolin with caveolae-associated proteins. J. Biol. Chem..

[37] Leonoudakis D., Conti L.R., Radeke C.M., McGuire L.M., Vandenberg C.A. (2004). A multiprotein trafficking complex composed of SAP97, CASK, Veli, and Mint1 is associated with inward rectifier Kir2 potassium channels. J. Biol. Chem..

[38] Pan C.Q., Sudol M., Sheetz M., Low B.C. (2012). Modularity and functional plasticity of scaffold proteins as p(l)acemakers in cell signaling. Cell. Signal..

[39] Steele D.F., Eldstrom J., Fedida D. (2007). Mechanisms of cardiac potassium channel trafficking. J. Physiol..

[40] Ma D., Zerangue N., Lin Y.F., Collins A., Yu M., Jan Y.N., Jan L.Y. (2001). Role of ER export signals in controlling surface potassium channel numbers. Science.

[41] Zuzarte M., Rinne S., Schlichthorl G., Schubert A., Daut J., Preisig-Muller R. (2007). A di-acidic sequence motif enhances the surface expression of the potassium channel TASK-3. Traffic.

[42] Martinez-Marmol R., Perez-Verdaguer M., Roig S.R., Vallejo-Gracia A., Gotsi P., Serrano-Albarras A., Bahamonde M.I., Ferrer-Montiel A., Fernandez-Ballester G., Comes N. (2013). A non-canonical di-acidic signal at the C-terminus of Kv1.3 determines anterograde trafficking and surface expression. J. Cell Sci..

[43] Spear J.M., Koborssy D.A., Schwartz A.B., Johnson A.J., Audhya A., Fadool D.A., Stagg S.M. (2015). Kv1.3 contains an alternative C-terminal ER exit motif and is recruited into COPII vesicles by Sec24a. BMC Biochem..

[44] Chen L., Jeffries O., Rowe I.C., Liang Z., Knaus H.G., Ruth P., Shipston M.J. (2010). Membrane trafficking of large conductance calcium-activated potassium channels is regulated by alternative splicing of a transplantable, acidic trafficking motif in the RCK1-RCK2 linker. J. Biol. Chem..

[45] Levitan E.S., Takimoto K. (2000). Surface expression of Kv1 voltage-gated K+ channels is governed by a C-terminal motif. Trends Cardiovasc. Med..

[46] Akhavan A., Atanasiu R., Noguchi T., Han W., Holder N., Shrier A. (2005). Identification of the cyclic-nucleotide-binding domain as a conserved determinant of ion-channel cell-surface localization. J. Cell Sci..

[47] Michelsen K., Schmid V., Metz J., Heusser K., Liebel U., Schwede T., Spang A., Schwappach B. (2007). Novel cargo-binding site in the beta and delta subunits of coatomer. J. Cell Biol..

[48] Zerangue N., Schwappach B., Jan Y.N., Jan L.Y. (1999). A new ER trafficking signal regulates the subunit stoichiometry of plasma membrane K(ATP) channels. Neuron.

[49] Kupershmidt S., Yang T., Chanthaphaychith S., Wang Z., Towbin J.A., Roden D.M. (2002). Defective human Ether-a-go-go-related gene trafficking linked to an endoplasmic reticulum retention signal in the C terminus. J. Biol. Chem..

[50] Zuzarte M., Heusser K., Renigunta V., Schlichthorl G., Rinne S., Wischmeyer E., Daut J., Schwappach B., Preisig-Muller R. (2009). Intracellular traffic of the K+ channels TASK-1 and TASK-3: Role of N- and C-terminal sorting signals and interaction with 14-3-3 proteins. J. Physiol..

[51] Manganas L.N., Wang Q., Scannevin R.H., Antonucci D.E., Rhodes K.J., Trimmer J.S. (2001). Identification of a trafficking determinant localized to the Kv1 potassium channel pore. Proc. Natl. Acad. Sci. USA.

[52] Gomez-Posada J.C., Etxeberria A., Roura-Ferrer M., Areso P., Masin M., Murrell-Lagnado R.D., Villarroel A. (2010). A pore residue of the KCNQ3 potassium M-channel subunit controls surface expression. J. Neurosci..

[53] Zarei M.M., Eghbali M., Alioua A., Song M., Knaus H.G., Stefani E., Toro L. (2004). An endoplasmic reticulum trafficking signal prevents surface expression of a voltage- and Ca2+-activated K+ channel splice variant. Proc. Natl. Acad. Sci. USA.

[54] Sole L., Roig S.R., Vallejo-Gracia A., Serrano-Albarras A., Martinez-Marmol R., Tamkun M.M., Felipe A. (2016). The C-terminal domain of Kv1.3 regulates functional interactions with the KCNE4 subunit. J. Cell Sci..

[55] Li H., Guo W., Mellor R.L., Nerbonne J.M. (2005). KChIP2 modulates the cell surface expression of Kv 1.5-encoded K(+) channels. J. Mol. Cell. Cardiol..

[56] Etxeberria A., Aivar P., Rodriguez-Alfaro J.A., Alaimo A., Villace P., Gomez-Posada J.C., Areso P., Villarroel A. (2008). Calmodulin regulates the trafficking of KCNQ2 potassium channels. FASEB J..

[57] Alaimo A., Gomez-Posada J.C., Aivar P., Etxeberria A., Rodriguez-Alfaro J.A., Areso P., Villarroel A. (2009). Calmodulin activation limits the rate of KCNQ2 K+ channel exit from the endoplasmic reticulum. J. Biol. Chem..

[58] Liu W., Devaux J.J. (2014). Calmodulin orchestrates the heteromeric assembly and the trafficking of KCNQ2/3 (Kv7.2/3) channels in neurons. Mol. Cell. Neurosci..

[59] Cavaretta J.P., Sherer K.R., Lee K.Y., Kim E.H., Issema R.S., Chung H.J. (2014). Polarized axonal surface expression of neuronal KCNQ potassium channels is regulated by calmodulin interaction with KCNQ2 subunit. PLoS ONE.

[60] Joiner W.J., Khanna R., Schlichter L.C., Kaczmarek L.K. (2001). Calmodulin regulates assembly and trafficking of SK4/IK1 Ca2+-activated K+ channels. J. Biol. Chem..

[61] Balut C.M., Hamilton K.L., Devor D.C. (2012). Trafficking of intermediate (KCa3.1) and small (KCa2.x) conductance, Ca(2+)-activated K(+) channels: A novel target for medicinal chemistry efforts?. ChemMedChem.

[62] Hund T.J., Mohler P.J. (2011). Differential roles for SUR subunits in KATP channel membrane targeting and regulation. Am. J. Physiol. Heart Circ. Physiol..

[63] Li X., Ortega B., Kim B., Welling P.A. (2016). A Common Signal Patch Drives AP-1 Protein-dependent Golgi Export of Inwardly Rectifying Potassium Channels. J. Biol. Chem..

[64] Jensen C.S., Watanabe S., Rasmussen H.B., Schmitt N., Olesen S.P., Frost N.A., Blanpied T.A., Misonou H. (2014). Specific sorting and post-Golgi trafficking of dendritic potassium channels in living neurons. J. Biol. Chem..

[65] Lim S.T., Antonucci D.E., Scannevin R.H., Trimmer J.S. (2000). A novel targeting signal for proximal clustering of the Kv2.1 K+ channel in hippocampal neurons. Neuron.

[66] Rivera J.F., Ahmad S., Quick M.W., Liman E.R., Arnold D.B. (2003). An evolutionarily conserved dileucine motif in Shal K+ channels mediates dendritic targeting. Nat. Neurosci..

[67] Perez-Verdaguer M., Capera J., Martinez-Marmol R., Camps M., Comes N., Tamkun M.M., Felipe A. (2016). Caveolin interaction governs Kv1.3 lipid raft targeting. Sci. Rep..

[68] Bundis F., Neagoe I., Schwappach B., Steinmeyer K. (2006). Involvement of Golgin-160 in cell surface transport of renal ROMK channel: Co-expression of Golgin-160 increases ROMK currents. Cell. Physiol. Biochem..

[69] Taneja T.K., Ma D., Kim B.Y., Welling P.A. (2018). Golgin-97 Targets Ectopically Expressed Inward Rectifying Potassium Channel, Kir2.1, to the *trans*-Golgi Network in COS-7 Cells. Front. Physiol..

[70] Stockklausner C., Klocker N. (2003). Surface expression of inward rectifier potassium channels is controlled by selective Golgi export. J. Biol. Chem..

[71] Pan Z., Kao T., Horvath Z., Lemos J., Sul J.Y., Cranstoun S.D., Bennett V., Scherer S.S., Cooper E.C. (2006). A common ankyrin-G-based mechanism retains KCNQ and NaV channels at electrically active domains of the axon. J. Neurosci..

[72] Rasmussen H.B., Frokjaer-Jensen C., Jensen C.S., Jensen H.S., Jorgensen N.K., Misonou H., Trimmer J.S., Olesen S.P., Schmitt N. (2007). Requirement of subunit co-assembly and ankyrin-G for M-channel localization at the axon initial segment. J. Cell Sci..

[73] Hajdu P., Martin G.V., Chimote A.A., Szilagyi O., Takimoto K., Conforti L. (2015). The C-terminus SH3-binding domain of Kv1.3 is required for the actin-mediated immobilization of the channel via cortactin. Mol. Biol. Cell.

[74] Burke N.A., Takimoto K., Li D., Han W., Watkins S.C., Levitan E.S. (1999). Distinct structural requirements for clustering and immobilization of K+ channels by PSD-95. J. Gener. Physiol..

[75] Martens J.R., O’Connell K., Tamkun M. (2004). Targeting of ion channels to membrane microdomains: Localization of KV channels to lipid rafts. Trends Pharmacol. Sci..

[76] Romanenko V.G., Fang Y., Byfield F., Travis A.J., Vandenberg C.A., Rothblat G.H., Levitan I. (2004). Cholesterol sensitivity and lipid raft targeting of Kir2.1 channels. Biophys. J..

[77] Campomanes C.R., Carroll K.I., Manganas L.N., Hershberger M.E., Gong B., Antonucci D.E., Rhodes K.J., Trimmer J.S. (2002). Kv beta subunit oxidoreductase activity and Kv1 potassium channel trafficking. J. Biol. Chem..

[78] Nystoriak M.A., Zhang D., Jagatheesan G., Bhatnagar A. (2017). Heteromeric complexes of aldo-keto reductase auxiliary KVbeta subunits (AKR6A) regulate sarcolemmal localization of KV1.5 in coronary arterial myocytes. Chem. Biol. Interact..

[79] Lefoulon C., Waghmare S., Karnik R., Blatt M.R. (2018). Gating control and K(+) uptake by the KAT1 K(+) channel leaveraged through membrane anchoring of the trafficking protein SYP121. Plant Cell Environ..

[80] Kilisch M., Lytovchenko O., Schwappach B., Renigunta V., Daut J. (2015). The role of protein-protein interactions in the intracellular traffic of the potassium channels TASK-1 and TASK-3. Pflugers Archiv.

[81] Michaelevski I., Chikvashvili D., Tsuk S., Singer-Lahat D., Kang Y., Linial M., Gaisano H.Y., Fili O., Lotan I. (2003). Direct interaction of target SNAREs with the Kv2.1 channel. Modal regulation of channel activation and inactivation gating. J. Biol. Chem..

[82] Nehring R.B., Wischmeyer E., Doring F., Veh R.W., Sheng M., Karschin A. (2000). Neuronal inwardly rectifying K(+) channels differentially couple to PDZ proteins of the PSD-95/SAP90 family. J. Neurosci..

[83] Leonoudakis D., Mailliard W., Wingerd K., Clegg D., Vandenberg C. (2001). Inward rectifier potassium channel Kir2.2 is associated with synapse-associated protein SAP97. J. Cell Sci..

[84] Leonoudakis D., Conti L.R., Anderson S., Radeke C.M., McGuire L.M., Adams M.E., Froehner S.C., Yates J.R., Vandenberg C.A. (2004). Protein trafficking and anchoring complexes revealed by proteomic analysis of inward rectifier potassium channel (Kir2.x)-associated proteins. J. Biol. Chem..

[85] Saheki Y., De Camilli P. (2017). Endoplasmic Reticulum-Plasma Membrane Contact Sites. Annu. Rev. Biochem..

[86] Rabouille C. (2017). Pathways of Unconventional Protein Secretion. Trends Cell Biol..

[87] Yoo J.S., Moyer B.D., Bannykh S., Yoo H.M., Riordan J.R., Balch W.E. (2002). Non-conventional trafficking of the cystic fibrosis transmembrane conductance regulator through the early secretory pathway. J. Biol. Chem..

[88] Fox P.D., Haberkorn C.J., Akin E.J., Seel P.J., Krapf D., Tamkun M.M. (2015). Induction of stable ER-plasma-membrane junctions by Kv2.1 potassium channels. J. Cell Sci..

[89] Deutsch E., Weigel A.V., Akin E.J., Fox P., Hansen G., Haberkorn C.J., Loftus R., Krapf D., Tamkun M.M. (2012). Kv2.1 cell surface clusters are insertion platforms for ion channel delivery to the plasma membrane. Mol. Biol. Cell.

[90] Takimoto K., Yang E.K., Conforti L. (2002). Palmitoylation of KChIP splicing variants is required for efficient cell surface expression of Kv4.3 channels. J. Biol. Chem..

[91] Wong W., Schlichter L.C. (2004). Differential recruitment of Kv1.4 and Kv4.2 to lipid rafts by PSD-95. J. Biol. Chem..

[92] Bao L., Hadjiolova K., Coetzee W.A., Rindler M.J. (2011). Endosomal KATP channels as a reservoir after myocardial ischemia: A role for SUR2 subunits. Am. J. Physiol. Heart Circ. Physiol..

[93] Gao Y., Balut C.M., Bailey M.A., Patino-Lopez G., Shaw S., Devor D.C. (2010). Recycling of the Ca2+-activated K+ channel, KCa2.3, is dependent upon RME-1, Rab35/EPI64C, and an N-terminal domain. J. Biol. Chem..

[94] Lee H.S., Qi Y., Im W. (2015). Effects of *N*-glycosylation on protein conformation and dynamics: Protein Data Bank analysis and molecular dynamics simulation study. Sci. Rep..

[95] Watanabe I., Zhu J., Recio-Pinto E., Thornhill W.B. (2004). Glycosylation affects the protein stability and cell surface expression of Kv1.4 but Not Kv1.1 potassium channels. A pore region determinant dictates the effect of glycosylation on trafficking. J. Biol. Chem..

[96] Thayer D.A., Yang S.B., Jan Y.N., Jan L.Y. (2016). N-linked glycosylation of Kv1.2 voltage-gated potassium channel facilitates cell surface expression and enhances the stability of internalized channels. J. Physiol..

[97] Zhu J., Yan J., Thornhill W.B. (2012). *N*-glycosylation promotes the cell surface expression of Kv1.3 potassium channels. FEBS J..

[98] Noma K., Kimura K., Minatohara K., Nakashima H., Nagao Y., Mizoguchi A., Fujiyoshi Y. (2009). Triple *N*-glycosylation in the long S5-P loop regulates the activation and trafficking of the Kv12.2 potassium channel. J. Biol. Chem..

[99] Napp J., Monje F., Stuhmer W., Pardo L.A. (2005). Glycosylation of Eag1 (Kv10.1) potassium channels: Intracellular trafficking and functional consequences. J. Biol. Chem..

[100] Mant A., Williams S., Roncoroni L., Lowry E., Johnson D., O’Kelly I. (2013). *N*-glycosylation-dependent control of functional expression of background potassium channels K2P3.1 and K2P9.1. J. Biol. Chem..

[101] Petrecca K., Atanasiu R., Akhavan A., Shrier A. (1999). N-linked glycosylation sites determine HERG channel surface membrane expression. J. Physiol..

[102] Gong Q., Anderson C.L., January C.T., Zhou Z. (2002). Role of glycosylation in cell surface expression and stability of HERG potassium channels. Am. J. Physiol. Heart Circ. Physiol..

[103] Brooks N.L., Corey M.J., Schwalbe R.A. (2006). Characterization of *N*-glycosylation consensus sequences in the Kv3.1 channel. FEBS J..

[104] Hall M.K., Weidner D.A., Bernetski C.J., Schwalbe R.A. (2014). N-Linked glycan site occupancy impacts the distribution of a potassium channel in the cell body and outgrowths of neuronal-derived cells. Biochim. Biophys. Acta.

[105] Hall M.K., Weidner D.A., Chen J., Bernetski C.J., Schwalbe R.A. (2013). Glycan structures contain information for the spatial arrangement of glycoproteins in the plasma membrane. PLoS ONE.

[106] Bas T., Gao G.Y., Lvov A., Chandrasekhar K.D., Gilmore R., Kobertz W.R. (2011). Post-translational *N*-glycosylation of type I transmembrane KCNE1 peptides: Implications for membrane protein biogenesis and disease. J. Biol. Chem..

[107] Yoo D., Fang L., Mason A., Kim B.Y., Welling P.A. (2005). A phosphorylation-dependent export structure in ROMK (Kir 1.1) channel overrides an endoplasmic reticulum localization signal. J. Biol. Chem..

[108] Martinez-Marmol R., Comes N., Styrczewska K., Perez-Verdaguer M., Vicente R., Pujadas L., Soriano E., Sorkin A., Felipe A. (2016). Unconventional EGF-induced ERK1/2-mediated Kv1.3 endocytosis. Cell. Mol. Life Sci..

[109] Hammond R.S., Lin L., Sidorov M.S., Wikenheiser A.M., Hoffman D.A. (2008). Protein kinase a mediates activity-dependent Kv4.2 channel trafficking. J. Neurosci..

[110] Lin L., Sun W., Wikenheiser A.M., Kung F., Hoffman D.A. (2010). KChIP4a regulates Kv4.2 channel trafficking through PKA phosphorylation. Mol. Cell. Neurosci..

[111] Yang J.W., Vacher H., Park K.S., Clark E., Trimmer J.S. (2007). Trafficking-dependent phosphorylation of Kv1.2 regulates voltage-gated potassium channel cell surface expression. Proc. Natl. Acad. Sci. USA.

[112] Nesti E., Everill B., Morielli A.D. (2004). Endocytosis as a mechanism for tyrosine kinase-dependent suppression of a voltage-gated potassium channel. Mol. Biol. Cell.

[113] Misonou H., Mohapatra D.P., Park E.W., Leung V., Zhen D., Misonou K., Anderson A.E., Trimmer J.S. (2004). Regulation of ion channel localization and phosphorylation by neuronal activity. Nat. Neurosci..

[114] Fernandez-Orth J., Ehling P., Ruck T., Pankratz S., Hofmann M.S., Landgraf P., Dieterich D.C., Smalla K.H., Kahne T., Seebohm G. (2017). 14-3-3 Proteins regulate K2P 5.1 surface expression on T lymphocytes. Traffic.

[115] Bogeski I., Niemeyer B.A. (2014). Redox regulation of ion channels. Antioxid. Redox Signal..

[116] Svoboda L.K., Reddie K.G., Zhang L., Vesely E.D., Williams E.S., Schumacher S.M., O’Connell R.P., Shaw R., Day S.M., Anumonwo J.M. (2012). Redox-sensitive sulfenic acid modification regulates surface expression of the cardiovascular voltage-gated potassium channel Kv1.5. Circ. Res..

[117] Chimote A.A., Kuras Z., Conforti L. (2012). Disruption of kv1.3 channel forward vesicular trafficking by hypoxia in human T lymphocytes. J. Biol. Chem..

[118] Jindal H.K., Folco E.J., Liu G.X., Koren G. (2008). Posttranslational modification of voltage-dependent potassium channel Kv1.5: COOH-terminal palmitoylation modulates its biological properties. Am. J. Physiol. Heart Circ. Physiol..

[119] Jeffries O., Geiger N., Rowe I.C., Tian L., McClafferty H., Chen L., Bi D., Knaus H.G., Ruth P., Shipston M.J. (2010). Palmitoylation of the S0-S1 linker regulates cell surface expression of voltage- and calcium-activated potassium (BK) channels. J. Biol. Chem..

[120] Tian L., McClafferty H., Knaus H.G., Ruth P., Shipston M.J. (2012). Distinct acyl protein transferases and thioesterases control surface expression of calcium-activated potassium channels. J. Biol. Chem..

[121] Alioua A., Li M., Wu Y., Stefani E., Toro L. (2011). Unconventional myristoylation of large-conductance Ca(2)(+)-activated K(+) channel (Slo1) via serine/threonine residues regulates channel surface expression. Proc. Natl. Acad. Sci. USA.

[122] Lin D.H., Sterling H., Wang Z., Babilonia E., Yang B., Dong K., Hebert S.C., Giebisch G., Wang W.H. (2005). ROMK1 channel activity is regulated by monoubiquitination. Proc. Natl. Acad. Sci. USA.

[123] Albesa M., Grilo L.S., Gavillet B., Abriel H. (2011). Nedd4-2-dependent ubiquitylation and regulation of the cardiac potassium channel hERG1. J. Mol. Cell. Cardiol..

[124] Jespersen T., Membrez M., Nicolas C.S., Pitard B., Staub O., Olesen S.P., Baro I., Abriel H. (2007). The KCNQ1 potassium channel is down-regulated by ubiquitylating enzymes of the Nedd4/Nedd4-like family. Cardiovasc. Res..

[125] Cao Q., Zhong X.Z., Zou Y., Zhang Z., Toro L., Dong X.P. (2015). BK Channels Alleviate Lysosomal Storage Diseases by Providing Positive Feedback Regulation of Lysosomal Ca2+ Release. Dev. Cell.

[126] Cang C., Aranda K., Seo Y.J., Gasnier B., Ren D. (2015). TMEM175 Is an Organelle K(+) Channel Regulating Lysosomal Function. Cell.

[127] Quesada I., Rovira J.M., Martin F., Roche E., Nadal A., Soria B. (2002). Nuclear KATP channels trigger nuclear Ca(2+) transients that modulate nuclear function. Proc. Natl. Acad. Sci. USA.

[128] Stonehouse A.H., Grubb B.D., Pringle J.H., Norman R.I., Stanfield P.R., Brammar W.J. (2003). Nuclear immunostaining in rat neuronal cells using two anti-Kir2.2 ion channel polyclonal antibodies. J. Mol. Neurosci..

[129] Chachi L., Shikotra A., Duffy S.M., Tliba O., Brightling C., Bradding P., Amrani Y. (2013). Functional KCa3.1 channels regulate steroid insensitivity in bronchial smooth muscle cells. J. Immunol..

[130] Li B., Jie W., Huang L., Wei P., Li S., Luo Z., Friedman A.K., Meredith A.L., Han M.H., Zhu X.H. (2014). Nuclear BK channels regulate gene expression via the control of nuclear calcium signaling. Nat. Neurosci..

[131] Jang S.H., Byun J.K., Jeon W.I., Choi S.Y., Park J., Lee B.H., Yang J.E., Park J.B., O’Grady S.M., Kim D.Y. (2015). Nuclear localization and functional characteristics of voltage-gated potassium channel Kv1.3. J. Biol. Chem..

[132] Katta S.S., Smoyer C.J., Jaspersen S.L. (2014). Destination: Inner nuclear membrane. Trends Cell Biol..

[133] Chen Y., Sanchez A., Rubio M.E., Kohl T., Pardo L.A., Stuhmer W. (2011). Functional K(v)10.1 channels localize to the inner nuclear membrane. PLoS ONE.

[134] Soltysinska E., Bentzen B.H., Barthmes M., Hattel H., Thrush A.B., Harper M.E., Qvortrup K., Larsen F.J., Schiffer T.A., Losa-Reyna J. (2014). KCNMA1 encoded cardiac BK channels afford protection against ischemia-reperfusion injury. PLoS ONE.

[135] Szabo I., Bock J., Grassme H., Soddemann M., Wilker B., Lang F., Zoratti M., Gulbins E. (2008). Mitochondrial potassium channel Kv1.3 mediates Bax-induced apoptosis in lymphocytes. Proc. Natl. Acad. Sci. USA.

[136] Yu K.Y., Wang Y.P., Wang L.H., Jian Y., Zhao X.D., Chen J.W., Murao K., Zhu W., Dong L., Wang G.Q. (2014). Mitochondrial KATP channel involvement in angiotensin II-induced autophagy in vascular smooth muscle cells. Basic Res. Cardiol..

[137] Singh H., Lu R., Bopassa J.C., Meredith A.L., Stefani E., Toro L. (2013). MitoBK(Ca) is encoded by the Kcnma1 gene, and a splicing sequence defines its mitochondrial location. Proc. Natl. Acad. Sci. USA.

[138] Foster D.B., Ho A.S., Rucker J., Garlid A.O., Chen L., Sidor A., Garlid K.D., O’Rourke B. (2012). Mitochondrial ROMK channel is a molecular component of mitoK(ATP). Circ. Res..

[139] De Marchi U., Sassi N., Fioretti B., Catacuzzeno L., Cereghetti G.M., Szabo I., Zoratti M. (2009). Intermediate conductance Ca2+-activated potassium channel (KCa3.1) in the inner mitochondrial membrane of human colon cancer cells. Cell Calcium.

[140] Dolga A.M., Netter M.F., Perocchi F., Doti N., Meissner L., Tobaben S., Grohm J., Zischka H., Plesnila N., Decher N. (2013). Mitochondrial small conductance SK2 channels prevent glutamate-induced oxytosis and mitochondrial dysfunction. J. Biol. Chem..

[141] Testai L., Barrese V., Soldovieri M.V., Ambrosino P., Martelli A., Vinciguerra I., Miceli F., Greenwood I.A., Curtis M.J., Breschi M.C. (2016). Expression and function of Kv7.4 channels in rat cardiac mitochondria: Possible targets for cardioprotection. Cardiovasc. Res..

[142] Yao J., McHedlishvili D., McIntire W.E., Guagliardo N.A., Erisir A., Coburn C.A., Santarelli V.P., Bayliss D.A., Barrett P.Q. (2017). Functional TASK-3-Like Channels in Mitochondria of Aldosterone-Producing Zona Glomerulosa Cells. Hypertension.

[143] Smith C.O., Wang Y.T., Nadtochiy S.M., Miller J.H., Jonas E.A., Dirksen R.T., Nehrke K., Brookes P.S. (2018). Cardiac metabolic effects of KNa1.2 channel deletion and evidence for its mitochondrial localization. FASEB J..

[144] Villarejo A., Buren S., Larsson S., Dejardin A., Monne M., Rudhe C., Karlsson J., Jansson S., Lerouge P., Rolland N. (2005). Evidence for a protein transported through the secretory pathway en route to the higher plant chloroplast. Nat. Cell Biol..

[145] Huang C.Y., Chiang S.F., Lin T.Y., Chiou S.H., Chow K.C. (2012). HIV-1 Vpr triggers mitochondrial destruction by impairing Mfn2-mediated ER-mitochondria interaction. PLoS ONE.

[146] Von Charpuis C., Meckel T., Moroni A., Thiel G. (2015). The sorting of a small potassium channel in mammalian cells can be shifted between mitochondria and plasma membrane. Cell Calcium.

[147] Vicente R., Villalonga N., Calvo M., Escalada A., Solsona C., Soler C., Tamkun M.M., Felipe A. (2008). Kv1.5 association modifies Kv1.3 traffic and membrane localization. J. Biol. Chem..

[148] Manganas L.N., Trimmer J.S. (2000). Subunit composition determines Kv1 potassium channel surface expression. J. Biol. Chem..

[149] Zhu J., Watanabe I., Gomez B., Thornhill W.B. (2003). Heteromeric Kv1 potassium channel expression: Amino acid determinants involved in processing and trafficking to the cell surface. J. Biol. Chem..

[150] Ponce-Balbuena D., Guerrero-Serna G., Valdivia C.R., Caballero R., Diez-Guerra F.J., Jimenez-Vazquez E.N., Ramirez R.J., Monteiro da Rocha A., Herron T.J., Campbell K.F. (2018). Cardiac Kir2.1 and NaV1.5 Channels Traffic Together to the Sarcolemma to Control Excitability. Circ. Res..

[151] Utrilla R.G., Nieto-Marin P., Alfayate S., Tinaquero D., Matamoros M., Perez-Hernandez M., Sacristan S., Ondo L., de Andres R., Diez-Guerra F.J. (2017). Kir2.1-Nav1.5 Channel Complexes Are Differently Regulated than Kir2.1 and Nav1.5 Channels Alone. Front. Physiol..

